# How Accurate is Web-Based Self-Reported Height, Weight, and Body Mass Index in Young Adults?

**DOI:** 10.2196/jmir.2909

**Published:** 2014-01-07

**Authors:** Kirrilly Pursey, Tracy L Burrows, Peter Stanwell, Clare E Collins

**Affiliations:** ^1^Faculty of Health and MedicinePriority Research Centre for Physical Activity and NutritionUniversity of NewcastleCallaghanAustralia; ^2^Faculty of Health and MedicinePriority Research Centre for Translational Neuroscience and Mental HealthUniversity of NewcastleCallaghanAustralia

**Keywords:** Internet, height, weight, body mass index, self-report

## Abstract

**Background:**

Web-based approaches are an effective and convenient medium to deliver eHealth interventions. However, few studies have attempted to evaluate the accuracy of online self-reported weight, and only one has assessed the accuracy of online self-reported height and body mass index (BMI).

**Objective:**

This study aimed to validate online self-reported height, weight, and calculated BMI against objectively measured data in young Australian adults.

**Methods:**

Participants aged 18-35 years were recruited via advertisements on social media sites and reported their current height and weight as part of an online survey. They then subsequently had the same measures objectively assessed by a trained researcher.

**Results:**

Self-reported height was significantly overestimated by a mean of 1.36 cm (SD 1.93; *P*<.001), while self-reported weight was significantly underestimated by –0.55 kg (SD 2.03; *P*<.001). Calculated BMI was also underestimated by –0.56 kg/m^2^ (SD 0.08; *P*<.001). The discrepancy in reporting resulted in the misclassification of the BMI category of three participants. Measured and self-reported data were strongly positively correlated (height: *r*=.98, weight: *r*=.99, BMI: *r*=.99; *P*<.001). When accuracy was evaluated by BMI category and gender, weight remained significantly underreported by females (*P*=.002) and overweight/obese participants (*P*=.02).

**Conclusions:**

There was moderate to high agreement between self-reported and measured anthropometric data. Findings suggest that online self-reported height and weight can be a valid method of collecting anthropometric data.

## Introduction

Web-based approaches are becoming an increasingly popular and effective medium to collect epidemiological data and deliver eHealth interventions [1,2]. Web-based delivery is more cost effective than face-to-face interaction [3], can improve access to services for those in rural and remote locations, and allows provider contact with a large number of people simultaneously [4]. Online data collection and delivery of programs is also convenient with materials accessible at any time online, allowing for participation at times that are more opportune or outside regular hours [5].

To be effective, data that are self-reported via eHealth studies need to be reliable and accurate so that a participant’s health status can be assessed and progress can be monitored. Discrepancies between measured and self-reported anthropometric data can lead to a misclassification of weight status and can thus affect assessment of participant health. Therefore the validation of self-reported Web-based data is essential.

Previous research indicates that a variety of factors including gender, age, and body mass index (BMI) can affect the accuracy of paper-based and interview-based self-reported anthropometric data [6,7]. There is a tendency for height to be overestimated and weight and BMI to be underestimated [8-12]. This leads to a subsequent misclassification of BMI category as a result of misreported anthropometric data [7,8,13], which is significant given that BMI is a commonly used indicator of health status in epidemiological research. Given that these differences exist, it is likely that similar differences may exist between online self-report and measured data; however to date, the latter has not been well explored.

Self-reported data are subject to influence by factors including social desirability and mode of data collection, leading to estimation bias of anthropometric data [14]. Social norms to conform to a certain body ideal can affect reporting of anthropometric data [15], with one study reporting the classification of more individuals as obese in face-to-face interviews compared to via telephone interviews [16]. Mail-in surveys are associated with more accurate reporting of anthropometric data because participants are not as likely to be affected by the social pressures associated with data collection via interview. Similar to mail-in surveys, it may be assumed that the anonymity of Web-based data collection may result in more accurate self-reporting compared to face-to-face and telephone interviews. However, in a study conducted by Lassalle et al [17], reporting bias of Web-based self-reported anthropometric data was similar to that observed in face-to-face interviews. Therefore, the level of reporting bias associated with Web-based self-reporting of anthropometric data must be studied to determine its accuracy and appropriateness as a method of data collection.

To the authors’ knowledge, very few studies have assessed online self-reported data. One study that recruited adult participants (N=2513) found significant underreporting of online self-reported weight by –0.49 kg and overreporting of online self-reported height by 0.56 cm. This resulted in the significant underreporting of BMI in the study (*P<*.05). In addition, Bonn et al found significant underreporting of weight (mean difference -1.2 kg, SD 2.6), although they validated online self-reported weight alone [18] without validating online self-reported height or BMI. Similarly, a study conducted by Harvey-Berino [3] found significant underreporting of weight (mean difference -0.86 kg). However, this study was set in the context of a weight loss intervention, which could have potentially made them more aware of their current weight, that is, they were less likely to have misreported their data, although that could not be assessed. Additionally, the study sample population were all in the overweight/obese category (mean BMI 35.6 m/kg^2^, SD 6.5, range 25-50).

No studies have attempted to validate online self-reported height, weight, and BMI data in a young adult population. To the best of our knowledge, the current study is the first to evaluate accuracy of Web-based self-reported height compared to measured height in the young adult population in all weight categories. This has allowed for the calculation and comparison of BMI from self-reported and measured data in the youth population. The aim of this study was to validate self-reported height, weight, and calculated BMI data via an online survey compared to objectively measured data in young Australian adults.

## Methods

### Participants

Males and females living in New South Wales, Australia, aged 18-35 years were recruited from March-May 2013 via a media release coordinated by the University of Newcastle, Australia, including advertisements on the university website, online blog, and “virtual snowballing” using social media sites including Facebook. Participants were excluded if they were currently pregnant or not currently living in Australia. This study was conducted as part of a 164-item online food addiction survey that took approximately 30 minutes to complete. The survey included questions investigating perceptions of food addiction, demographic details, anthropometric data, and current dietary habits. As part of the survey, respondents were asked to self-report their current height and weight via the online tool, SurveyMonkey [19]. Demographic information was collected including gender, age, education, and postal code. Socioeconomic status was determined using the Socio-Economic Indexes for Areas (SEIFA) deciles, whereby postal areas receive a score of 1 to 10, with the lowest 10% of areas given the score of 1 and the highest 10% of areas are given the score of 10. Self-reported BMI was calculated from online self-reported height and weight using the standard equation, weight/height^2^ (kg/m^2^).

Upon completion of the survey, participants were invited to attend a voluntary anthropometric measurement session on campus at the University of Newcastle and were contacted via email to select their preferred time via the online scheduling link, Doodle [20]. Within 1 month of completing the survey, body measurements were taken by a trained assessor using a standardized protocol. Participant height was measured to 0.1 cm by the BSM370 Stadiometer, and verbal instructions were provided according to the stretch stature method [21]. Weight, fat mass, and fat free mass were measured to 0.1 kg using the InBody720 bioelectrical impedance analyzer with shoes and heavy clothing removed. BMI was calculated using the same equation used for the calculation of self-reported BMI, and participants were subsequently classified as underweight (<18.49 kg/m^2^), healthy weight (18.0-24.99 kg/m^2^), overweight (25-29.99 kg/m^2^) or obese (>30 kg/m^2^) using the World Health Organization cut points [22]. At the end of the session, participants were provided with personalized feedback of their results relative to normative standards. Written informed consent was obtained from all participants prior to measurement. This study was approved by the University of Newcastle Human Research Ethics committee.

### Statistics

Participant characteristics were checked for normality and analyzed descriptively, with mean (standard deviation) reported. Paired *t* tests were used to evaluate differences between self-reported and measured data. Pearson correlation was used to examine the strength of linear relationships between self-reported and measured data. To further investigate the relationship between variables, a multiple regression model using age, gender, and BMI was used. The degree of agreement between self-reported and measured data was also assessed using Bland-Altman plots [23]. Cohen’s *d* was used to compare effect sizes across different measures [24] and allowed for a more direct comparison of intervention effects on each outcome variable. These were calculated using the mean difference and the pooled standard deviation of the group (*d*=M_1_-M_2_ / σ_pooled_). Respondents were grouped and analyzed by age (18-25 years or >25-35 years), gender, and BMI category (healthy weight or overweight/obese) to determine differences between self-reported and measured data both within groups and across groups. Due to the small sample sizes, overweight and obese participants were grouped and analyzed together and underweight participants were excluded from analysis. Statistics were computed using Stata V12. Significance level was set at .05.

## Results

A total of 504 participants completed the broader food addiction survey, with n=117 in the validation study (23.2%). Participant characteristics are described in Table 1. Participants were predominantly female (79.5%, 93/117) with mean age 23.74 years (SD 3.92, range 18-35) and were from a range of socioeconomic backgrounds (35% SEIFA 5-6 deciles). The most commonly reported highest level of education achieved by the participants was a high school certificate (46.2%, 54/117) followed by a university degree (29.1%, 34/117). Mean BMI calculated from measured data was 24.18 kg/m^2^ (SD 5.62, range 16.3-53) with the majority of participants classified as healthy weight (73.5%, 86/117). Three participants were classified as underweight, 16 as overweight, and 12 as obese. BMI calculated using self-reported data did not change BMI classification significantly with 5 participants classified as underweight, 87 as healthy weight, 13 as overweight, and 12 as obese. There were no significant differences between study participants and nonparticipants (n=367) of the larger survey with respect to demographic variables and self-reported height and weight (*P*>.05).

**Table 1:** Baseline data of adults participating in the Web-based food addiction study.

Characteristics		Male	Female	Total
Participants (n)		24	93	117
Age (years), mean (SD)		24.54 (3.57)	23.45 (4.54)	23.74 (3.92)
**SEIFA** ^a^ **deciles, n (%)**				
	1-2 (lowest)	0 (0.0)	4 (4.3)	4 (3.4)
	3-4	5 (20.8)	23 (24.7)	28 (23.9)
	5-6	6 (25.0)	35 (37.6)	41 (35.0)
	7-8	5 (20.8)	15 (16.1)	20 (17.1)
	9-10 (highest)	8 (33.3)	16 (17.2)	24 (20.5)
**BMI** ^b^ **category (kg/m** ^**2**^ **), n (%)**				
	Underweight	0 (0.0)	3 (3.2)	3 (2.6)
	Healthy weight	19 (79.2)	67 (72.0)	86 (73.5)
	Overweight	4 (16.7)	12 (12.9)	16 (13.7)
	Obese	1 (4.2)	11 (11.8)	12 (10.2)
Fat mass (kg), mean (SD)		11.20 (8.87)	20.33 (13.28)^c^	18.43 (13.00)
Fat free mass (kg), mean (SD)		65.20 (9.02)	46.18 (6.34)^d^	50.10 (10.34)
Body fat (%), mean (SD)		13.89 (6.85)	28.58 (9.38)^d^	25.48 (10.63)

^a^SEIFA: socioeconomic index for areas

^b^BMI: body mass index; weight (kg) / height (m)^2^

^c^ *P=*.002

^d^ *P<*.001

**Figure 1:**
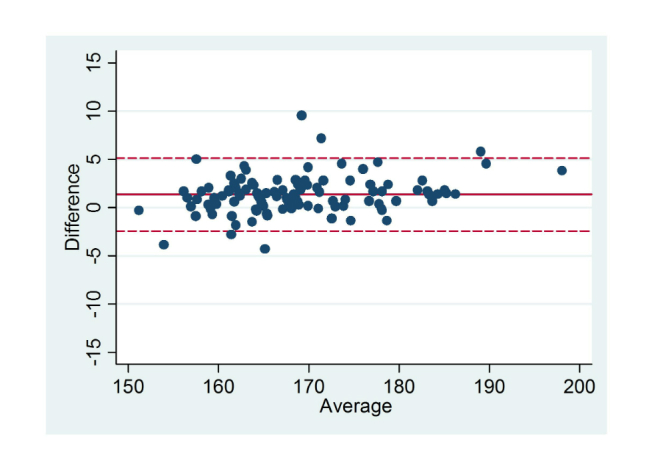
Level of agreement between self-reported and measured height (cm). Solid line represents the mean difference and dotted line represents the limits of agreement (LOA).

**Figure 2:**
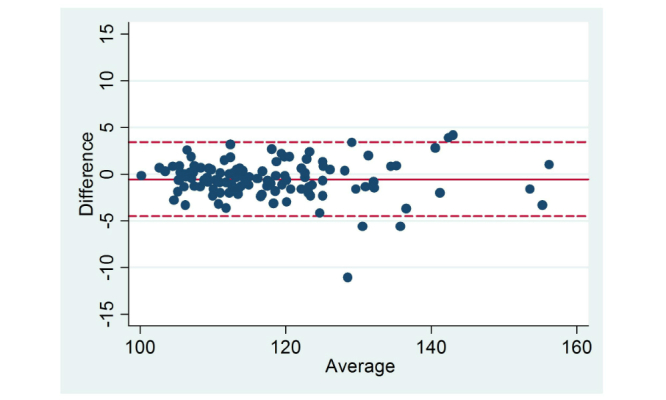
Level of agreement between self-reported and measured weight (kg). Solid line represents the mean difference and dotted line represents the limits of agreement (LOA).

**Figure 3:**
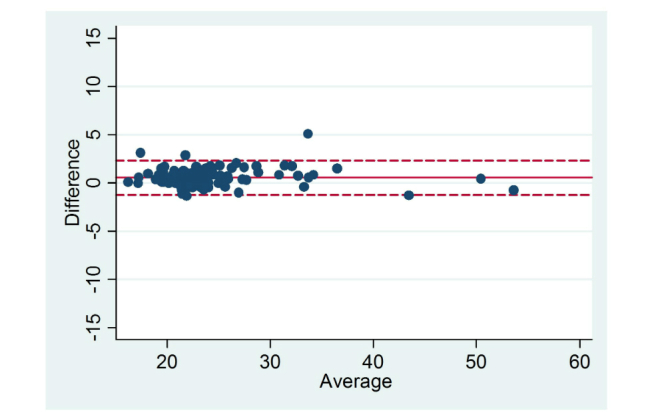
Level of agreement between BMI calculated from self-reported and measured data (kg/m^2^). Solid line represents the mean difference and dotted line represents the limits of agreement (LOA).

Differences in self-reported and measured data as well as effect sizes are reported in Tables 2 and 3. Mean self-reported height, 169.35 cm (SD 8.86) was significantly higher than measured height (mean 167.99 cm, SD 8.37; mean difference 1.36 cm, SD 1.93, *P<*.001). Self-reported weight (mean 67.93 kg, SD 17.39) was significantly lower than measured weight (mean 68.48 kg, SD 17.59; mean difference –0.55 kg, SD 2.03, *P=*.004). As a result of the discrepancies between self-reported and measured height and weight, BMI calculated from self-reported height and weight was significantly lower than measured BMI (mean –0.56 kg/m^2^, SD 0.08, *P<*.001). Self-reported height and weight and calculated BMI were highly correlated with the corresponding measured data (height: *r*=.98, weight: *r*=.99, BMI: *r*=.99; *P<*.001). Figures 1-3 display the Bland Altman plots for the average versus mean difference in self-reported and actual measurements. The limits of agreement (LOA) were wide for each variable of height, weight, and BMI. At the group level, the majority of values fell within the LOA (2SD) indicating a fairly good level of agreement. In descending order, the mean difference (LOA) when compared to measured data for each variable was –0.55 kg (–4.61, 3.51) for weight, 0.56 kg/m^2^ (–1.26, 2.37) for BMI, and 1.37 cm (–2.49, 5.22) for height. Analysis using Cohen’s *d* showed there was no or little effect (Cohen’s *d*≤.50) on all variables (height, weight, BMI); range was 0.01-0.30. Despite the lower number of male participants, effect sizes were generally higher for males than females.

When grouped by BMI category, self-reported and measured weight did not differ significantly in healthy weight participants (*P=*.07) but remained significantly underreported in overweight/obese participants (*P=*.02). Discrepancies between self-reported and measured weight and BMI were significant between those overweight/obese compared to healthy weight (*P=*.02 and *P=*.03 respectively). Self-reported weight was significantly underreported by females (*P=*.002) but not by males (*P=*.71). Differences between self-reported and measured height were significant for males and females (*P=*.02 and *P=*.03 respectively). When grouped by age category, individuals aged 18-25 years were found to significantly underreport weight (*P=*.02), but not in individuals >25 years (*P=*.06). Height and BMI remained significantly misreported for all groups when grouped by BMI, age, and gender. When controlling for variables including gender, age, and BMI, the relationship between self-reported and measured data for each outcome measure remained highly significant (*P<*.001). However, these additional explanatory variables did not increase the strength of the associations.

**Table 2:** Differences between Web-based self-reported and measured height (cm) and weight (kg) in adults (n=117) grouped by BMI, age, and gender.

		Self-reported height	Measured height	Difference	*P* ^ a^	*d^ b^*	Self-reported weight	Measured weight	Difference	*P* ^ a^	*d^ b^*
		Mean (SD)	Mean (SD)	Mean (SD)			Mean (SD)	Mean (SD)	Mean (SD)		
All		169.35 (8.86)	167.99 (8.37)	1.36 (1.93)	<.001	0.16	67.93 (17.39)	68.48 (17.59)	0.55 (2.03)	.004	0.03
**Gender**									
	Male (n=24)	180.48 (7.52)	178.69 (6.94)	1.79 (1.71)	<.001	0.25	76.13 (13.81)	76.31 (14.72)	–0.18 (2.38)	.71	0.01
	Female (n=93)	166.50 (6.56)	165.20 (6.21)	1.29 (2.02)	<.001	0.20	65.82 (17.70)	66.48 (17.80)	–0.60 (1.91)	.002	0.04
	*P* value between groups				.23					.32	
**Age (years)**									
	18-25 (n=87)	169.11 (8.82)	167.76 (8.36)	1.35 (1.99)	<.001	0.15	65.74 (16.02)	66.22 (16.00)	–0.50 (2.00)	.028	0.03
	>25-35 (n=30)	170.05 (9.08)	168.63 (8.50)	1.42 (1.75)	<.001	0.17	74.30 (19.82)	75.04 (20.45)	–0.74 (2.06)	.058	0.03
	*P* value between groups				.88					.55	
**BMI^c^ (kg/m** ^**2**^ **)**									
	Healthy weight (n=86)	169.04 (9.10)	167.63 (8.56)	1.41 (2.05)	<.001	0.16	61.81 (7.71)	62.12 (7.51)	–0.31 (1.63)	.08	0.04
	Overweight/Obese (n=28)	170.89 (8.30)	169.59 (8.00)	1.30 (1.63)	<.001	0.16	89.16 (21.63)	90.52 (21.40)	–1.36 (2.97)	.02	0.06
	*P* value between groups				.80					.02	

^a^ *P* value

^b^Cohen's *d*

^c^BMI: body mass index; weight (kg) / height (m)^2^

**Table 3:** Differences between Web-based self-reported and measured BMI (kg/m^2^) in adults (n=117) grouped by BMI, age, and gender.

		Self-reported BMI, mean (SD)	Measured BMI, mean (SD)	Difference, mean (SD)	Cohen’s *d*
All		23.63 (5.60)	24.18 (5.62)	–0.56 (0.08)^a^	0.11
**Gender**					
	Male (n=24)	23.30 (3.38)	23.80 (3.66)	0.51 (0.97)^b^	0.14
	Female (n=93)	23.75 (6.18)	24.31 (6.06)	0.57 (0.89)^a^	0.10
	*P* value between groups			.78	
**Age (years)**					
	18-25 (n=87)	22.94 (5.21)	23.46 (5.17)	0.52 (0.91)^a^	0.12
	>25-35 (n=30)	25.63 (6.29)	26.28 (6.40)	0.65 (0.89)^a^	0.11
	*P* value between groups			.51	
**BMI** ^c^ **(kg/m** ^**2**^ **)**					
	Healthy weight (n=86)	21.60 (1.74)	22.06 (1.58)	–0.46 (0.77)^a^	0.30
	Overweight/Obese (n=28)	30.57 (7.56)	31.46 (7.26)	–0.89 (1.22)^a^	0.12
	*P* value between groups			.03	

^a^ *P<*.001

^b^ *P=*.02

^c^BMI: body mass index; weight (kg) / height (m)^2^

## Discussion

### Principal Findings

To our knowledge, this is the first study to evaluate the accuracy of online self-reported height and weight in a young adult population. Compared to objectively measured data, online self-reported height was significantly overestimated while weight was significantly underestimated. As a consequence of the differences in self-reported and measured height and weight, self-reported BMI was significantly underestimated by participants; this underestimation of BMI changed the classification of BMI category of three participants. Measured and self-reported height, weight, and BMI were all strongly positively correlated with moderate levels of agreement. When grouped by BMI, age, and gender, self-reported weight remained significantly underreported by females, overweight/obese participants, and individuals <25 years. Effect sizes in this study (Cohen’s *d*) for subgroups were considered small and likely to reflect the overall small sample size.

There was fairly good agreement between self-reported and measured data in the current study. Thus, online self-reported height and weight can be accepted as a satisfactory method of data collection in Web-based weight interventions, which is in agreement with international studies [3,17,18]. The discrepancies between self-reported and measured weight in the current study were smaller than those reported by two previous online studies validating weight alone [3,18] but were greater than those reported by a third larger study that also assessed height and BMI [17]. In addition, misreporting of anthropometric data in the current study related to BMI classification [17] and gender bias [18] is consistent with previous research in the area. These discrepancies in online self-reported data highlight that the same medium should be used to collect data for repeated measures within trials.

### Strengths and Limitations

The generalizability of the current study may be limited by the recruitment of a convenience sample of predominantly female participants who were interested in a survey about food addiction. Those who volunteered to be measured were participants in an online food addiction survey where the only incentive was providing personalized feedback regarding height, weight, and body composition. Thus it is possible that these individuals may be more motivated than the general population, and the smaller magnitude of differences in the current study compared to previous paper-based self-reported studies [9-12] could be evidence that measurements may be affected by volunteer bias. However, the participants that were measured were representative of the larger online survey sample.

Another limitation of the study is the time lapse of 1 month between self-report and measurement. Logistical issues related to time taken to recruit participants and accessing measurement facilities resulted in a longer period of time between self-report and measurement than previously conducted studies. This time-lag between self-report and measurement could potentially be enough time for weight to have changed. This is particularly important in college-aged participants whose weight has been shown to fluctuate rapidly [25] or could be enough time for weight to change if an individual was participating in a weight loss program. It is possible that the instructions given to the participants in self-reporting data, the clothing worn by participants at time of measurement, and the use of different measuring equipment by participants compared to the calibrated equipment used by the trained assessor could have introduced measurement bias [8]. However, we would expect to see systematic and larger differences between self-reported and measured data than the results obtained if differences were due to measurement error.

Strengths of the study include the use of measured height to validate calculated BMI and the inclusion of adults from all weight categories to allow for comparison of self-reported anthropometric data based on weight status. The current study is important as it includes individuals from a range of weight status categories, including healthy weight.

### Conclusions

Self-reported height was significantly overestimated and self-reported weight significantly underestimated by Australian adults aged 18-35 years. However, there was fairly good agreement between self-reported and measured data, and these were strongly positively correlated. When grouped by BMI category and demographic data, self-reported weight remained significantly underreported by individuals classified as overweight/obese, females, and individuals <25 years only. These findings suggest that online self-reported height and weight can be a valid method of collecting anthropometric data and calculating BMI. Future studies with larger sample sizes and repeated measures over time in eHealth research contexts are required.

## Abbreviations

BMI: body mass index
LOA: limits of agreement
SEIFA: Socio-Economic Indexes for Areas
